# A review of the International Seabed Authority database DeepData from a biological perspective: challenges and opportunities in the UN Ocean Decade

**DOI:** 10.1093/database/baad013

**Published:** 2023-03-30

**Authors:** M Rabone, T Horton, D O B Jones, E Simon-Lledó, A G Glover

**Affiliations:** Deep-Sea Systematics and Ecology Research Group, Life Sciences Department, Natural History Museum, Cromwell Rd, London SW7 5BD, UK; Ocean Biogeosciences, National Oceanography Centre, European Way, Southampton SO14 3ZH, UK; Ocean Biogeosciences, National Oceanography Centre, European Way, Southampton SO14 3ZH, UK; Ocean Biogeosciences, National Oceanography Centre, European Way, Southampton SO14 3ZH, UK; Deep-Sea Systematics and Ecology Research Group, Life Sciences Department, Natural History Museum, Cromwell Rd, London SW7 5BD, UK

## Abstract

There is an urgent need for high-quality biodiversity data in the context of rapid environmental change. Nowhere is this need more urgent than in the deep ocean, with the possibility of seabed mining moving from exploration to exploitation, but where vast knowledge gaps persist. Regions of the seabed beyond national jurisdiction, managed by the International Seabed Authority (ISA), are undergoing intensive mining exploration, including the Clarion–Clipperton Zone (CCZ) in the Central Pacific. In 2019, the ISA launched its database ‘DeepData’, publishing environmental (including biological) data. Here, we explore how DeepData could support biological research and environmental policy development in the CCZ (and wider ocean regions) and whether data are findable, accessible, interoperable and reusable (FAIR). Given the direct connection of DeepData with the regulator of a rapidly developing potential industry, this review is particularly timely. We found evidence of extensive duplication of datasets; an absence of unique record identifiers and significant taxonomic data–quality issues, compromising FAIRness of the data. The publication of DeepData records on the OBIS ISA node in 2021 has led to large-scale improvements in data quality and accessibility. However, limitations in the usage of identifiers and issues with taxonomic information were also evident in datasets published on the node, stemming from mismapping of data from the ISA environmental data template to the data standard Darwin Core prior to data harvesting by OBIS. While notable data-quality issues remain, these changes signal a rapid evolution for the database and significant movement towards integrating with global systems, through the usage of data standards and publication on the global data aggregator OBIS. This is exactly what has been needed for biological datasets held by the ISA. We provide recommendations for the future development of the database to support this evolution towards FAIR.

**Database URL** https://data.isa.org.jm/isa/map

## Introduction

The need for high-quality biodiversity data is abundantly clear in the face of the biodiversity crisis, with numerous pressures impacting species, including climate change (1). Such data are essential for understanding ecosystems, detecting and monitoring anthropogenic impacts and developing effective environmental policy. To be usable for both research and policy, it is important that data meet the criteria of being findable, accessible, interoperable and reusable (FAIR) (2). For example, FAIR biodiversity information can be fed into frameworks for monitoring and observation, such as essential ocean variables (EOVs) and essential biodiversity variables (EBVs), and utilized in environmental policy (3, 4). However, major gaps in coverage of global biodiversity data across thematic and geographical areas have been identified (5, 6). Further, the biodiversity data landscape is highly heterogeneous, with varying degrees of data integration and exchange (7–10). This landscape is also characterized by a multitude of databases, some highly specialized, by theme, region, taxon or similar (e.g. Fishbase; www.fishbase.org) and some broad, global aggregators, e.g. the Global Biodiversity Information Facility (GBIF; https://www.gbif.org).

**Figure 1.**
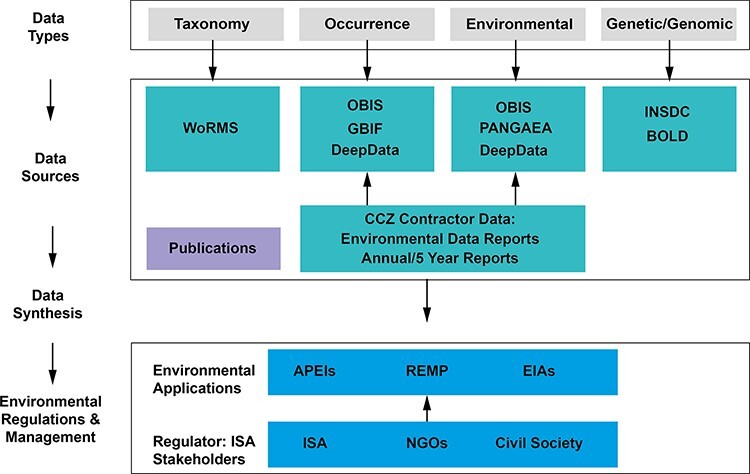
The Clarion-Clipperton Zone biodiversity data landscape, showing relevant key data types: taxonomy, occurrence, environmental and genetic/genomic data; key data sources: databases, publications and contractor data; and how these data, once synthesized in publications and meta-analyses, could contribute to environmental management applications, with input by the regulator, the ISA and wider stakeholders. Key databases as listed: WoRMS, OBIS, GBIF, PANGAEA (Data Publisher for Earth & Environmental Science), INSDC and BOLD (Barcode Of Life Data System). Environmental applications: APEIs, REMP and EIAs (environmental impact assessments).

Relevant data types in biodiversity include taxonomy, occurrence, environmental and genetic/genomic data (8; Figure 1). Biological databases often specialize by data type, e.g. the World Register of Marine Species (WoRMS; https://www.marinespecies.org) is focused on taxonomy, as a checklist and classification infrastructure for marine taxa (11–13). They also exchange information, e.g. the global ocean data aggregator Ocean Biodiversity Information System (OBIS; https://obis.org/) specializing in marine occurrence and environmental data utilizes the WoRMS taxonomic backbone (14, 15). Global data standards such as Darwin Core (DwC) administered by Biodiversity Information Standards [formerly Taxonomic Databases Working Group (TDWG); www.tdwg.org] allow for data interoperability and exchange (16). In addition to data standards such as DwC, there are many relevant standardization efforts. For example, the Ocean Best Practices System under the auspices of the Intergovernmental Oceanographic Commission provides a platform for best practices with a ‘semantic’ approach, linking relevant protocols (17–19). However, adoption of standards and best practices is variable (8, 20). Key challenges include treatment of taxonomic information—a long-standing issue in biology (11, 12, 21)—and problems with validity of identifiers, compromising data exchange, traceability and contributing to duplication (7, 20, 22–25).

Nowhere are these challenges more apparent than for deep-sea biodiversity data, where most species are undescribed (11, 26, 27). Extensive usage of informal (or ‘temporary’) taxa names given to species prior to formal description (see 28; 29, 30) or ‘open nomenclature’ *sensu* Horton *et al.* (11) and Sigovini *et al.* (31) compound the existing challenges in taxonomy. The open ocean and the deep sea represent key information gaps in global biodiversity data coverage (5, 32, 33). However, regions of the deep ocean are undergoing intensive exploration for mining of polymetallic nodules, in particular the Clarion–Clipperton Zone in the Central Pacific. Mineral-related activities in the deep seabed beyond national jurisdictions are managed by the International Seabed Authority (ISA), a body established under the United Nations Convention on the Law of the Sea. As part of the mineral exploration process, the ISA requires the holders of exploration contracts to collect and make available environmental and biological data to improve the understanding of deep-sea ecosystems and the impacts of potential deep-sea mining activities (34).

The need for a central ISA database was formally identified by the Legal and Technical Commission (LTC) of the ISA in 2002 (ISBA/8/C/6). Several LTC recommendations were made during the period of 2002–2019 (ISBA/8/C/6; ISBA/5/C/6; ISBA/21/C/16; ISBA/22/LTC/15), and DeepData was developed as a further iteration of the previous Central Data Repository, which was primarily focused on mineral resources. In 2019, the ISA launched the public database DeepData as a repository of deep-seabed-related data collected by contractors and related parties (e.g. research organizations conducting surveys) in the Area (https://data.isa.org.jm/isa/map). The database holds both geological data, categorized as confidential, and publicly available environmental data, an umbrella term for environmental and biological data in ISA parlance. DeepData is unusual in the respect that there is a direct connection of the database with the regulator and that the main data providers to the database are contractors undertaking exploration of mineral resources in the Area, albeit working directly with the scientific community. The microcosm of the CCZ data landscape, however, illustrates general processes of how biological data types are collated and subsequently published from a range of sources (Figure 1). Further, given the direct connection of the regulator and database, it also illustrates how these data could be synthesized and applied to environmental management, e.g. in developing tools such as the regional environmental management plan (REMP) or the design of areas of particular environmental interest (APEIs; see Figure 1 and Smith et al. (35)).

In this study, we provide the first review of DeepData, focused on biological data available for the most active area of seabed mining (CCZ), and include recommendations for the development of this database into the future. This work is particularly timely, given DeepData has now been operational for four years; associated records are being actively pushed onto global data aggregators such as OBIS, GBIF and International Nucleotide Sequence Collaboration (INSDC) and OBIS is also now publishing DeepData records via the OBIS ISA node (https://obis.org/node/9d2d95be-32eb-4d81-8911-32cb8bc641c8). More crucially however, is the rapid recent development of deep-seabed mining regulations and the urgent need to address deep-sea biodiversity data gaps both for the CCZ and other regions (36, 37). The United Nations (UN) Ocean Decade has also resulted in a renewed focus on the importance and usability of ocean data (38). Here, we conduct an assessment of the database and wider related ISA biological/environmental data management as part of a broader study where we synthesize the biodiversity and biogeographic data available from DeepData and associated databases for the CCZ (39); Rabone et al., Accepted. The primary purpose of this study therefore is to assess the FAIRness of published biological data in DeepData and the potential utility of the database to support both research and decision-making for environmental policy.

## Materials and methods

### Overview of DeepData and description of the online data portal

The ISA DeepData website or online data portal provides biological, envionmental, geochemical and physical data collated from expeditions arranged by contractors for the CCZ and other exploration regions. The map-based interface includes boundary data (e.g. shapefiles) depicting APEIs, mining exploration contract areas, reserved mining exploration areas and research sample data. The datasets currently held in the database include biological and geochemical analyses from samples collected using box corers, epibenthic sledges, multiple corers, remotely operated vehicles (ROVs) and benthic trawls; navigational information from expeditions; current meter recordings and water column and water sampling data. The DeepData web interface has two windows: ‘HOME’ with a map view and ‘MAP OPTIONS’ with six tabs ‘Layers’, ‘Search’, ‘CTD’, ‘Photo/Video Gallery’, ‘Library’ and ‘Docs’ on the left-hand side, with the map on the right (Figure S1; https://data.isa.org.jm/isa/map/). Options to select biological data by category, on the ‘MAP OPTIONS’ window, ‘Layers’ tab are as follows: ‘Contractors – Mineral Type’ [here, contractors are listed by mineral type (cobalt-rich ferromanganese crust (CRFC)/polymetallic nodules (PMNs)/polymetallic sulphides (PMSs)), with separate entries for the same contractor holding contracts in different mineral types ]; ‘Contract Status’ (all/active/extended); ‘Sponsoring State’; ‘Mineral Type’ (CRFC/PMN/PMS) and ‘Location’ [including Central Indian Ocean/Central Indian Ridge and Southeast Indian Ridge/CCFZ/Indian Ocean/Indian Ocean Ridge/Mid-Atlantic Ridge/Rio Grande Rise/South Atlantic Ocean/Southwest Indian Ridge/Variable—PMN Reserved Areas/Western Pacific Ocean]. Options to search and download data are on the adjacent ‘Search’ tab, and under ‘filter by data type’ is a dropdown menu to select first data type: ‘Biological’ or ‘Environmental Chemistry’, and second sampling method: ‘Point’ or ‘Trawl Line’ (Figure S1). Here, ‘Point’ equates to deployments (sampling events) collected from a particular point in space and time—e.g. a box core—and ‘Trawl Line’ equivalent to those collected from sampling between two points—e.g. via ROVs or towed gear such as a Brenke epibenthic sledge trawl sample.

### Data collection

Biological data were downloaded from the DeepData database web portal on 12 July 2021. The data selection was conducted as follows: ‘Layers’ tab: ‘Mineral Type’: ‘Polymetallic Nodules’, ‘Location’: ‘Clarion Clipperton Fracture Zone’, Search tab: ‘Biological data’, ‘Point’, and to export the data, ‘export query’ (File S1A). The same search procedure was run again with the other ‘Biological Data’ option available: ‘Trawl Line’ (File S1B). For contextual spatial data, all mining exploration contract areas, both active and reserved, and APEI shapefiles were downloaded from the ISA database (https://www.isa.org.jm/minerals/maps) and combined into one shapefile in Quantum GIS (QGIS, version 3.10; A Coruña; QGIS.org, 2020). Co-ordinates for a polygon covering the CCZ including the combined shapefile were established: (in decimal degrees, longitude/latitude): northwest −164.01462, 15.70629; southwest −155.04998, –5.51238; southeast −101.9181, 6.05623 and northeast −117.66088, 23.72549. DeepData records have been harvested by OBIS and published on the OBIS ISA node since June 2021 (available at https://obis.org/node/9d2d95be-32eb-4d81-8911-32cb8bc641c8). OBIS occurrence data were downloaded as a DwC file on 12 July 2021 using the ‘occurrence’ function in the robis package (Provoost & Bosch, 2017), with the CCZ polygon as delineated above, for all depths. Data were not downloaded direct from the node to allow consistency with data collection from other databases for the parallel study (39; Rabone et al., Accepted).

### Data processing and analysis

#### Data restructuring and general data processing

Data were processed and analysed in R, version 4.0.2 (2020-06-22), ‘Taking Off Again’ (R Core Team, 2020). General quantitative and qualitative observations as well as structured notes were made for analysis. Preliminary investigations of the database export showed that the records (or observations) were distributed both across columns and rows, rather than one record per row (40). The data were restructured to one record per row using the ‘spread’ function in R from the tidyverse package (41). The separate ‘Point’ and ‘Trawl Line’ data downloads were combined into the same dataset (File S1C). As the data fields varied between the two datasets—e.g. ‘actual latitude’ in ‘Point’ data and ‘startLatitude’ and ‘endLatitude’ in ‘Trawl Line’ data—fields were harmonized. For co-ordinates and depth, the end-point was used, i.e. ‘endLatitude’ was mapped to ‘actualLatitude’, to allow the datasets to be combined (File S1C, D). Initial assessments of the data revealed that the database export file did not contain a record identifier or a unique key in any format, primary, composite or other. To examine the data, a composite key was created, combining the DeepData identifier fields for the contractor, station and specimen (‘ContractorID’ + ‘StationID’ + ‘SampleID’). The key was checked for duplicates, and none were found. Data columns were checked and edited where necessary (e.g. for depth, missing values were listed as −9, and these were replaced with ‘NA’). Where possible this was scripted in R, where multiple entries for character variables were present, this was done in Microsoft Excel 365 on a copy of the data column, renamed with the suffix ‘_ed’ (File S1C, D).

#### Geographic mapping

Contractor sub-areas were mapped in QGIS, and the data were revised to reflect actual administrative areas (i.e. contract area or APEI), rather than the origin of records (i.e. ContractorID: name of contractor submitting data) as these were not equivalent. All OBIS records were mapped together with the CCZ shapefile, using the following R packages: GADMTools (https://github.com/IamKDO/GADMTools); sp and spData (Pebesma EJ, Bivand RS (2005). Classes and methods for spatial data in R. R News, 5 (2), 9–13. https://CRAN.R-project.org/doc/Rnews/); spatialEco (Evans, J.S. (2021). spatialEco. R package version 1.3–6, https://github.com/jeffreyevans/spatialEco); maptools (http://maptools.r-forge.r-project.org/); rgdal (http://rgdal.r-forge.r-project.org) and rgeos (https://r-forge.r-project.org/projects/rgeos/). The records were then sub-selected by depth, with depths of 3000 m and greater included. Some records without depth values were present, those falling within or near the CCZ shapefile were reviewed and included if valid, for example if a benthic species associated with a publication and a benthic collection method e.g. a box core sample; and/or a relevant reference in ‘datasetName’ or ‘associatedReferences’ column. The DeepData records published on OBIS were sub-selected from general OBIS records (distinguished as recorded as owned by the ISA in the DwC ‘accessRights’ field; File S2).

#### Taxonomic data

Initial examination of taxonomic information found extensive inconsistent recording of names, e.g. misspellings, misformatting, e.g. escaped newlines and misrecording, e.g. class names recorded in the Family field. No DwC equivalent field for ‘scientificName’ was present, i.e. the lowest taxonomic-level identification of the specimen referenced in a given record (see List of Terms). To allow data to be analysed for the parallel study (39; Rabone et al., Accepted), this field was added for all records, populated with the lowest taxonomic-level identification present per record. If a name was noted with question mark, recorded with a qualifier indicating uncertainty in identification, e.g. sp. inc. or *Incerta,* or written as two names, the next highest taxonomic level recorded was added as the scientific name. For example, if two family names were present, indicating a level of uncertainty in the identification, then the order was recorded as the scientific name. Preliminary investigations showed significant numbers of informal species names (temporary names) and/or ‘open nomenclature’ designations, e.g. names recorded with qualifiers, such as cf. (11, 31). Where open nomenclature designations were provided, a scientific name was also recorded, mapped to the lowest taxonomic-level identification above the species level. If a species name (i.e. specific epithet) was present, then the genus name only was recorded in the scientificName field. The taxonomic information was cleaned using ‘taxonMatch’ in WoRMS, a QA/QC (quality assurance/quality control) function on the website where scientific names can be validated against the database (www.marinespecies.org). Resulting names were cross-referenced, any usage of unaccepted names was recorded and corresponding accepted names were added to the newly created ‘scientificName’ field. If no match was found on WoRMS, the original name was retained. Any qualifiers recorded with a name, e.g. ‘cf.’, were mapped to a separate identification qualifier field, and the taxonomic level of the qualifier was recorded. A sample of contractor data submissions was requested from the ISA for insight into both ISA data mapping processes and contractor data recording. A selection of records from six contractors from annual data reporting submissions from 2015 to 2017 were provided, and datasets were harmonized and processed into one file (File S3). Structured notes were made on data quality for taxonomy fields both for the published records and the unprocessed contractor data files, for general context and comparison.

## Results

### Data structure of database export

The data export from DeepData of biological ‘Point’ data from the 12th of July consisted of a dataset of dimensions: 981 483 rows and 48 columns. Post–data restructuring to one observation per row resulted in a file of 52 177 rows and 56 columns. The data export of ‘Trawl Line’ data consisted of a much smaller dataset of 941 rows and 49 columns, restructured to 45 rows. The two files were then combined to produce a final dataset of 52 222 rows and 56 columns (File S1C). As the parallel study was examining benthic metazoans only, records of non-metazoans, such as xenophyophores, or records without taxonomic information were removed. This resulted in a final dataset for analysis encompassing 40 518 rows and 56 columns (File S1D; also used in the parallel study, i.e. Rabone et al., Accepted). The distinction between ‘Points’ and ‘Trawl Line’ for records in DeepData was incomplete, with trawl-collected records evident in the ‘Point’ dataset (File S1A–D). The ‘Trawl Line’ data in the database output contained a sole dataset of 45 records from a single dataset, but 8197 records in total would have fallen into a ‘Trawl Line’ classification (e.g. collected by an epibenthic sledge, benthic trawl, automated underwater vehicle or ROV). Altogether 99.5% of ‘Trawl Line’ data were incorrectly classified as ‘Point’ data (8152 of 8197 records). This distinction between sampling types necessitates additional data processing to recombine them. It is also inaccurate in the database, as ‘Point’ data appear to be the default category, regardless of the actual sampling method information present. More fundamentally, the distinction is unnecessary, as sampling method is recorded in a separate column.

The structure of the DeepData output had observations distributed both over rows and columns or in both ‘wide’ and ‘long’ formats (38). Wide format is where one record or observation is captured in one row, and the ‘long’ format’ is where one record or observation is split across multiple rows. All data were in wide format, until the fields were ‘Analysis’ and ‘Result’, where these data fields were ‘paired’, i.e. ‘Result’ data values pertain to the adjacent field ‘Analysis’, and these data were therefore structured in long format (Table S1). The field ‘Analysis’ is a list of column headings, e.g. ‘Taxonomist’ and ‘Taxonomist E-mail’. These headings originate from the environmental data template (File S4A, B) and are grouped by ‘Category’ field two columns to the left (e.g. for ‘Category’: ‘Taxonomist information’, column headings as recorded in ‘Analysis’ include ‘Taxonomist’, ‘Taxonomist E-mail’, etc.). The ‘Result’ field records the related data for the adjacent ‘Analysis’ field, e.g. ‘Taxonomist’ in the ‘Analysis’ column and ‘Not Reported’ in the ‘Result’ column. The Analysis and Result columns are therefore paired, while the remainder of the table is in ‘wide’ format. This is illustrated with a subset of data in Table S1. This structure, with observations distributed both across rows and columns, produces significant redundancy in the data (Table S1). Another export option is available, ‘export pivot query’, and this option has all data in wide format but was not used in analysis as during initial exploratory investigations, it appeared to be primarily for contrasting visual formatting only and export query was assumed to be the default format. The Secretariat has since confirmed the two options are to account for different formats of data types (including non-biological data). Here, the restructured export query data are equivalent to export pivot query data, i.e. redundancy is removed.

### Data quality in database export

#### Taxonomy

As the database export file lacked a field equivalent to the DwC term scientificName, i.e. the lowest taxonomic identification of a given record, interpretation of the identification from the available taxonomic data fields and mapping of this information to a newly created field was required. Scenarios such as this which necessitate the interpretation of the data by a third party should be avoided. The output also did not include a separate field for the identification qualifier, with this information only recorded in a notes field or the actual taxonomy field/column (e.g. ‘cf. Munnopsidae’). Extensive usage of unaccepted names, multiple names per field, misspellings and notes in taxonomic data fields was evident (85% of records). This is clearly illustrated with the Phylum field, which contained 74 different entries while 31 (extant) metazoan phyla are currently recognized. The lower the taxonomic rank, the more variable the data entries present. Examination and cross-referencing of unprocessed contractor files revealed that taxonomic information for all fields was published verbatim (or close-to) from contractor data submissions (File S3), with minimal data processing evident. Where data processing of taxonomy has occurred however, it appears to have caused additional complexities, even taxonomic designations being changed in at least one case. For example, records of the annelid *Monticellina* Laubier, 1961, were present in DeepData incorrectly as *Monticellina* Westblad, 1953 (Platyhelminthes), rather than *Monticellina* Laubier, 1961, accepted as *Kirkegaardia* Blake, 2016 (Annelida). In the contractor data submissions, the relevant record was evidently *Kirkegaardia* by comparison with the higher taxonomy columns, but the genus name was recorded as *Monticellina,* the unaccepted homonym. A taxon match for the genus *Monticellina* in WoRMS returns an ‘ambiguous match’ (a standard result for homonyms, pre-occupied names and similar) with the two options (*Monticellina* Westblad, 1953, and *Kirkegaardia* Blake, 2016). The DeepData name matching appears to have been carried out with reference to the lowest taxonomic level only, as the record was taxon matched to *Monticellina* Westblad, 1953 (i.e. the Platyhelminthes genus) rather than correctly to the annelid genus *Kirkegaardia* Blake, 2016, an error that would have been picked up if higher taxonomic ranks were cross-referenced.

#### General data quality and missing information

Several other fields also required cleaning and harmonizing where data should match a standard set of terms, i.e. a controlled vocabulary. For example, the DeepData field ‘SampleCollectionMethod’ had variable entries, including misspellings (e.g. multi core, MUC, Multi Corer, Multi-corer). Contractors have recorded these data in variable ways in the data templates (File S3), and like the taxonomic data, the entries had not been harmonized prior to publication. For some fields, the origin of the information present was not clear as it does not appear in the contractor templates (File S3). For example, in the field ‘HabitatType’, approximately half the DeepData records had habitat recorded as ‘water column’, but none of the corresponding contractor files had ‘water column’ recorded in the habitat field, or elsewhere (see File S3).

In addition, 90% of data overall were missing or incomplete for multiple fields. Missing data included key information, such as sampling method, which is critical information for analysis. Sampling method was only present in 44% of the records (18 003/40 518). Size class is key information for deep-sea surveys with faunal groupings generally distinguished by size (e.g. meiofauna, macrofauna and megafauna). Data on size class were often missing, despite being a required field in the data template (‘nominalSizeCategory’). Omission of information has also produced inaccuracies. For example, in the field ‘Identification Method’ for recording how taxa were identified, text entries were present as ‘Morphological’ or ‘DNA’ but not as a combined entry, i.e. ‘Morphological and DNA’. This data recording is an artefact of an earlier iteration of the data template, where only one method could be recorded in the field, and as a result, the omission produces an inaccurate picture of how identifications were made.

As a wider point, data from the majority of cruises are yet to be published on the database, as according to the ISA Secretariat, over 100 cruises have been carried out in the CCZ, but records from only 24 cruises, and 10 contractors in total published at the time of data download (Table 1; Rabone & Glover, in review). It is unclear if this is entirely due to a data backlog or if there are cases of active contractors who have not submitted data. While substantial data cleaning was required for taxonomy and to a lesser extent, sampling information, site data in contrast required minimal processing. Some anomalies were still evident; for example, in the contractor sub-area field, a number of cases were designated as ‘OA’ (outside area) but were within the claim of that contractor (File S1C, D). This field could be made more informative by recording the contract area or APEI the sample was collected from.

**Table 1.** Cruises by year, contractor and research vessel in records in DeepData, as published at the time of data download (12 July 2021)

Year	Contractor/s	Research vessel	Total cruises
2004	IFREMER	*L’Atalante*	1
2010	BGR	*R/V Sonne*	2
	KOREA	*R/V Onnuri*	
2011	COMRA	*Hai Yang Liu Hao*	2
	KOREA	*Kok*	
2012	BGR/IFREMER	*L’Atalante*	1
2013	BGR	*R/V Kilo Moana*	4
	COMRA	*Hai Yang Liu Hao*	
	KOREA	*R/V Onnuri*	
	UKSRL	*R/V Melville*	
2014	BGR	*R/V Kilo Moana*	3
	COMRA	*Hai Yang Liu Hao*	
	YUZH/IOM	*Yuzhmorgeologiya*	
2015	BGR1/GSR/IFREMER	*R/V Sonne*	4
	OMS/UKSR	*R/V Thomas G. Thompson*	
	GSR	*Mt. Mitchell*	
	YUZH	*Yuzhmorgeologiya*	
2016	BGR	*R/V Kilo Moana*	3
	BGR	*R/V Sonne*	
	YUZH	*Yuzhmorgeologiya*	
2017	COMRA	*Xiangyanghong 03*	2
	DORD	*R/V Kilo Moana*	
2018	KOREA	*KODOS1802*	1
2019	KOREA	*KODOS2019*	1
TOTAL	10	13	24

For joint expeditions, both contractor codes are listed (e.g. YUZH/IOM). Total cruises = total cruises per year, as per available data on the DeepData database. Records from 10 contractors were published on DeepData at the time of data download: BGR (Germany), COMRA (China), DORD (Japan), KOREA (Government of the Republic of Korea), GSR (Belgium), IFREMER (France), IOM Interoceanmetal Joint Organization, OMS (Singapore), UKSRL (UK Seabed Resources Limited; the UK) and JSC Yuzhmorgeologiya (YUZH; Russian Federation). There are 16 CCZ-based contractors in total, but 17 contracts (UKSRL holds two separate contracts), and a further two contractors holding licences outside the CCZ. The following are the six contractors that have active licences in the CCZ but do not have data published on DeepData at the time of this study: TOML (Tonga), NORI (Nauru), MARAWA (Kiribati), CIIC (Cook Islands), CMC (China) and a new contractor, Blue Minerals Jamaica Ltd. It is unclear if the lack of data from these contractors is due entirely to a backlog of data publishing or if no data have been submitted yet.

#### Duplication

We found ∼6000 duplicate records for the contractor BGR (Federal Institute for Geosciences and Natural Resources of Germany) and ∼4000 for UKSRL (UK Seabed Resources Ltd) in the database export. Duplicates were suspected in other contractor datasets, including KOREA (Government of the Republic of Korea) and IOM (Interoceanmetal Joint Organization), and were confirmed via an OBIS pipeline for identifying duplication in datasets (available in a GitHub notebook, https://iobis.github.io/notebook-duplicates/). We estimate overall duplication is approximately a quarter of the total records assessed (∼10 000 of 40 518). The exact number of duplicates could not be ascertained because of underlying issues with identifiers (detailed in the following paragraphs). This duplication appears to have arisen through a combination of issues in versioning of annual contractor data submissions and usage of identifiers. Looking first at versioning, multiple years of the annual data submissions have been published, but this has resulted in duplicates. The ISA has been publishing the annual contractor data submissions year by year, from 2015—the year the environmental data template was introduced—and plans to continue until to date (ISA Secretariat, pers. comm.). For the yearly data reports, these are either one-off data submissions, e.g. a standalone dataset for a particular cruise that is not then resubmitted the following year, or are iterative data submissions, where records are added to the previous year’s dataset and any updates added to the existing ones. The latter applies to the UKSRL and BGR annual data submissions for example. However, they have been handled as separate datasets rather than yearly updates, resulting in duplication.

The duplicates are primarily stemming from issues with identifiers. The database export lacks a record identifier (or primary key) and uses the specimen identifier field ‘SampleID’ to reconcile records (Sheldon Carter, pers. comm.). In theory, any records submitted year on year with the same SampleID should therefore be matched and associated data updated if changed. For 20% of the records (8181), SampleID values were not unique. For raw contractor data, 86% of the records for the subset of 2015–2017 data submissions had SampleIDs that were either absent or not unique (File S3). Records missing a sample ID are allocated one during data processing (Sheldon Carter, pers. comm.). This explains why all records in the database export had a sample ID value even when they were absent from the raw contractor data. Here, however, the possibility arises again for duplication. For example, in the BGR data, where no sample IDs were present, records from the 2015 template were allocated a sample ID when that dataset was uploaded, and then the same records allocated a different sample ID when the 2016 and 2017 data were uploaded and therefore appear on DeepData output as separate records, producing duplication.

### Data fields in DeepData export

Several fields that are not required were included in the database export. For example, backend database names were evident: ‘AreaKey’, ‘ClusterID’ and ‘BlockID’, and for the latter two, no data entries were present in any case. While the search was for polymetallic nodule data only, the output included fields for vents and sulphide deposits, including ‘HydrothermalActivity’ and ‘HydrothermalVentAge’ and ‘ExtensionPMSSite’. Additional fields were present for taxonomic information, e.g. ‘Subfamily’, the only sub- or super-taxonomic classification field included. Both the reason for its inclusion and the rules around its usage are unclear, as it has been used not for subfamily names but rather as a field to capture temporary species names, even though there are two separate fields for recording this in the output: ‘Putative.species.name.or.number’ and ‘Morphotype’. Here, the former field has been replaced by the latter (‘Morphotype’) in the 2022 template (File S4). This may be why both fields were present in the database export, and the ‘Morphotype’ field was blank.

### The ISA environmental data template

The structure of the 2022 environmental data template is split into separate tables by tab, e.g. ‘Point Sample’, ‘Towed Gear Sample’, ‘Chem_Results’ and ‘Biological_Results’. The previous template (2018) was structured with all the tabs (i.e. subtables) as one wide table. The restructuring into several tables has improved usability but has also introduced new issues, for example the separation of ‘Point Sample’ and ‘Towed Gear Sample’ tables. The separation of point and trawl data has been made to link biological and resource data in the database as it reflects the underlying structure in the database (ISA Secretariat, pers. comm.) but creates an extra processing step that should not be necessary, particularly, since sampling information is recorded in a specific field.

Examining data fields in the tab ‘Biological_Results’, the 2022 template now includes fields for scientific name and taxonomic identification qualifier, essential fields for capturing taxonomic identification. These are notable improvements, saving significant processing time. Other key fields are still absent, however, such as a record identifier field that is persistent and unique (equivalent to occurrenceID in DwC; see List of Terms) and as distinct from a specimen identifier, i.e. SampleID (equivalent to catalogNumber in DwC; see Recommendations: ‘Data management considerations'). Another key field missing from the template is an equivalent for the DwC field ‘basisOfRecord’ for designating record type, for example ‘machineObservation’ for an ROV-derived record or ‘preservedSpecimen’ for a specimen-based one. As in the database export, superfluous fields were present. ‘OrgNum’ for example is a required field (‘TaxaID’ in the previous template) but is an arbitrary number to provide a composite key for ISA data processing. It is therefore a backend column name and as such a redundant field that does not capture any existing data in contractor datasets. It also necessitates an additional processing step by contractors and has the potential to cause confusion. Subfamily is included, but as indicated for the database export file, this field is not necessary.

In addition, some field naming and accompanying definitions are potentially ambiguous. The field ‘Matrix Type’ (Point Sample or Towed Gear Sample tabs) for example is to capture material or sample type (with a definition provided: ‘biological sample, sediment or water unfiltered’), but usage of the term ‘Matrix’ rather than more intuitive wording such as ‘sample’ or ‘material’ is potentially confusing. A more critical example is ‘SampleID’, which has been interpreted in a variety of ways by contractors. In some datasets, SampleID was used for a batch of samples, equivalent to a deployment or sampling event ID, rather than for an individual specimen record as intended (File S4A, B). The current data template includes the field ‘StationID’ for recording station number, but this does not account for multiple samplings at a given station, and the template does not include a deployment or sampling event ID to capture this (see Recommendations). Some contractors do not use the SampleID field at all, but rather other fields, such as ‘voucherCode’. Similarly, the field ‘Morphotype’, which is intended to capture temporary species names, could be misinterpreted, as this term usually refers to megafauna identified solely by imagery, as opposed to other types of temporary names such as molecular operational taxonomic units (MOTUs), which the field is also supposed to capture. The relevant DwC field here is ‘taxonConceptID’, which captures all types of open nomenclature or informal species names (11; see List of Terms). Our overall assessment post-testing of the new template and examining contractor data submissions (Files S3 and S4) is that issues with usage are likely to continue in the new template without a significant reworking including the incorporation of rules for filling out required fields.

### OBIS ISA node and DeepData mapping to DwC

The publishing of DeepData records on the OBIS ISA node necessitated a process of mapping contractor data to DwC terms by the ISA data team (see File S5). The resulting data were processed by the OBIS Secretariat for publication on the OBIS ISA node, documented in a GitHub notebook (https://github.com/iobis/notebook-deepdata). This data processing was carried out on the datasets mapped to DwC, in JSON (JavaScript Object Notation) format, on the ISA server, not a DwC archive (DwC-A) of the DeepData database output itself (Figure S2). This process of data mapping to DwC by the ISA Secretariat has resulted in the inclusion of critical data fields that were previously missing (e.g. scientificName, occurrenceID and basisOfRecord). The DwC terms have been misinterpreted in some places however by the ISA data team, and mismapping of data template fields to DwC was evident. For example, basisOfRecord, a key DwC term for describing the record type (see List of Terms), has been populated entirely with the text entry ‘taxon’. Mapping to DwC terms overall is incomplete, with terms not being utilized where corresponding data are captured in the template, e.g. INSDC accession numbers could be mapped to the term ‘associatedSequences’ in DwC. Some of these fields would be helpful for tracing records and identifying duplication, given the lack of adequate record identifiers, e.g. the DwC term ‘datasetName’ would delineate a particular dataset, such as an annual contractor data submission, supporting accurate versioning.

**Figure 2.**
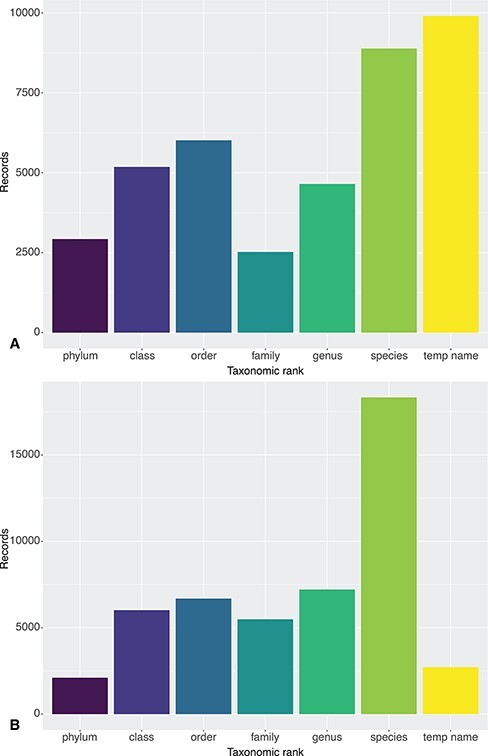
Taxonomic resolution of Clarion-Clipperton Zone DeepData records as published on 12 July 2021: (A) from DeepData itself and (B) via the OBIS ISA node. The record sets primarily differ in the number of temporary name records (9936 on DeepData and 2715 on OBIS) and species-level records (8883 on DeepData and 18 329 on OBIS). These differences are the result of mismapping of DwC by the ISA Secretariat, discussed in section 4.5. Note that temporary names here may include names at levels higher than species, i.e. temporary/informal species names (morphospecies), and also temporary names for higher taxon ranks, e.g. undescribed genera and incomplete identifications using open nomenclature. See Rabone et al. (2022) (39) for further discussion of informal names.

This misinterpretation of DwC terms has also produced incorrect taxonomic information. For example, ‘taxonConceptID’, a DwC field recommended by Horton et al. (2021) for recording of the open nomenclature name (or taxonomic concept) in DwC terms (11; see List of Terms), was incorrectly mapped to ‘taxonRemarks’. As a result, only 20% of the temporary name records in DeepData were present on the OBIS ISA node (2715/13 177 records; Figure 2). Additional issues have arisen during data processing for mapping to DwC, also impacting taxonomic information. In the process of mapping to ‘scientificName’, genus names have been duplicated in the resulting scientificName column and the duplicated genus names harvested instead of the species names, resulting in a much lower total number of species names on the OBIS ISA node, 75 compared to 466 (including pelagic species) from DeepData, as ascertained in the parallel study (39; Rabone et al., Accepted; Rabone & Glover, in review). The duplication of genus name also appears to have resulted in species names being reallocated to other phyla in at least one case. For example, records of the nematode *Capsula galeata* Bussau, 1993, (28 in total) were assigned to the diatom phylum Ocrophyta in the data mapping, presumably because scientificName was designated in DeepData as the genus name only, i.e. ‘*Capsula*’ rather than ‘*Capsula galataea*’; returning *Capsula* J. Brun, 1896†, an unassigned name in WoRMS. As a wider point, the DwC term taxonRank, which records the taxonomic level of scientific name, has been incorrectly populated, with the result that the number of records as ‘species’ (18 329) is almost double that of the actual total (Figure 2; Files S1C and S2).

Significant issues were also present in treatment of identifiers in the DwC mapping. The DwC term ‘occurrenceID’ is a key field for a persistent, unique record identifier and required for any data submission to OBIS or GBIF. Here, occurrenceID has been generated as a composite key, from combining ‘StationID’/‘TrawlID’ and ‘SampleID’. There were duplicates present in this composite key, however, 30% overall. These duplicates were also independently identified by the OBIS Secretariat and at the start of the OBIS processing pipeline records were allocated a separate unique identifier. Because of these duplicates in occurrenceID in the DeepData records, a proportion of records cannot be definitively matched between the two databases. Also, the occurrenceID as a non-unique composite key is not present in the DeepData export, only in the JSON files mapped to DwC (and therefore only in the OBIS ISA node records). The composite key would therefore need to be generated with the same formatting to allow any cross-referencing between the records from DeepData or OBIS, i.e. there is not a common record identifier. Even adding the composite key would not be sufficient for comparing the records of course as they do not match because the identifier is not unique. Further, as data processing for the records on DeepData differs from the records on the OBIS ISA node, the records appear different. Overall the number of records for benthic metazoans differed, 40 518 in DeepData and 48 536 in OBIS, which appears to be due in part to more datasets published on the OBIS ISA node than DeepData at the time of download, but this could not be clearly ascertained because of the underlying identifier issue and lack of dataset name in the DeepData records. In conclusion, standardization of data to DwC terms to prepare the DeepData records so that they can be harvested by OBIS has been a significant step forward, but incorrect data mapping in the process has also compromised data quality.

## Recommendations

The ISA has met a significant challenge to reconcile and publish often variable datasets from contractor annual environmental data submissions. It is a notable achievement that significant biological data holdings are now published and available on the database (>50 000 records and 40 518 for metazoans only at time of download). The 2022 template is also an improvement on the previous version. Through publishing of DeepData records on OBIS, and in the process, mapping data to DwC, some key issues have been addressed and the biological data can now, in part, be classified as FAIR (Table 2; File S7). Despite the issues detailed here, DeepData is a major step forward in developing a centralized repository of biological data in areas beyond national jurisdiction (ABNJs), especially given that there has only been four years of development since public release.

In a separate study, we have made the first attempt to survey all metazoan biodiversity data from the CCZ using DeepData and published species records (39; Rabone et al., Accepted). These kinds of regional syntheses would not be possible without the significant efforts from the ISA DeepData team. DeepData provides a crucial source of ‘raw’ occurrence data that are rarely available in publications, even as supplementary files, as revealed in the parallel study. A broader point is that the timing of this work has coincided with a phase of rapid evolution of the database and that the Secretariat is aware of the limitations discussed here and actively working to address them (ISA Secretariat, pers. comm.). There are significant improvements to be made, however, that can address the key data-quality issues, with the result of greater utility of the data.

**Figure 3.**
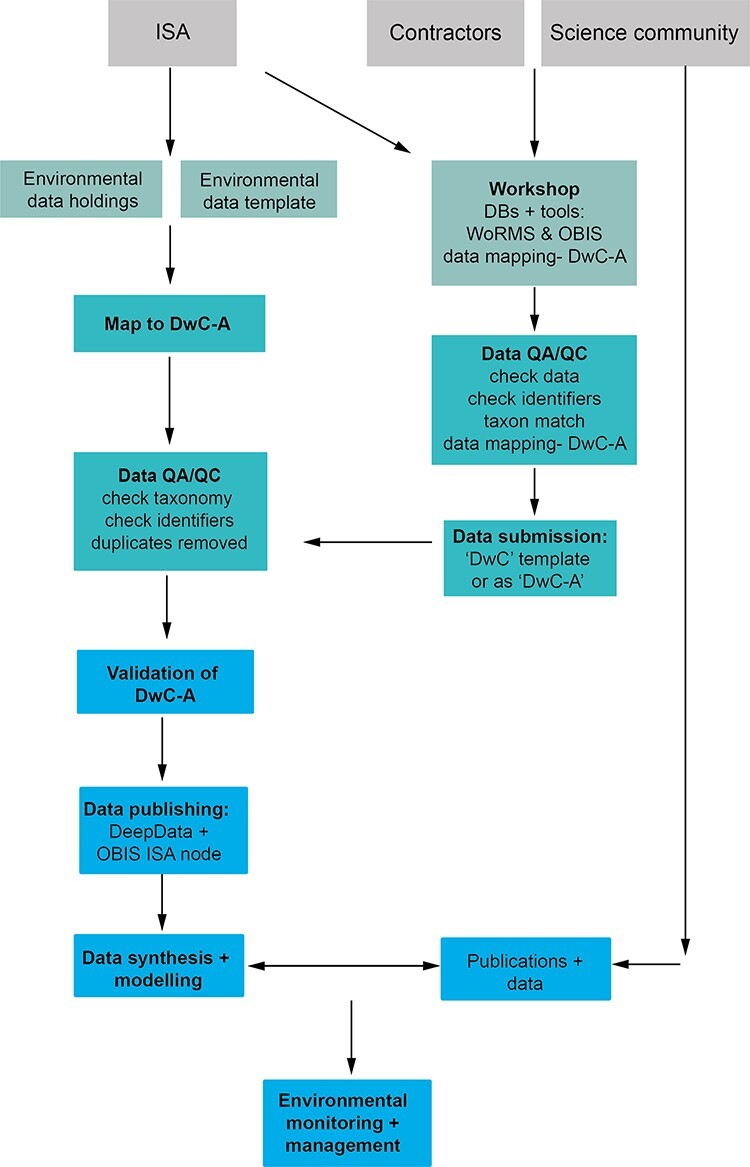
A proposed data management workflow for the ISA (processes shown in bold text). First, the current environmental contractor data template is replaced with a DwC compliant version with all fields (column headings) in DwC format, and contractors/data providers can alternatively submit data as a Darwin Core Archile (DwC-A) file. Existing environmental data holdings are remapped comprehensively to DwC terms as a batch process and undergo QA/QC prior to publication. Concurrently, a public workshop is delivered by the ISA with input from the contractors, the science community and other stakeholders (with full documentation available), covering DwC, databases, in particular WoRMS and OBIS, and tools such as taxon match in WoRMS and the GBIF DwC validator and assistant. Here, contractors undertake QA/QC checks and submit new data (in a new DwC compliant template or as a DwC-A). After QA/QC, datasets are published (as a DwC-A) on both DeepData and the OBIS ISA node. These data subsequently can be utilized for data synthesis and modelling and environmental policy applications.

It is important to note that the scope of our study is limited to biological data in the CCZ. DeepData is far from solely a biological database, and many other data types such as geochemistry, geology and wider environmental data are collected by contractors and held by the database. The FAIRness of these non-biological datasets should also be extensively reviewed. This is especially important given these data are only available through DeepData itself, and not also as Darwin Core published on OBIS. Geological data being confidential may be a complex case, but the potential for greater transparency should be explored as this would have significant scope for an improved understanding of ecosystems in the region. Here, we provide key recommendations with the aim of improving data quality for both research and environmental policy. These recommendations are also depicted as a potential workflow in Figure 3 and summarized in Table 2.

**Table 2.** Summary of DeepData assessment, including key current limitations, their implications and suggested solutions or recommendations to address these and source of good examples in global databases

Identified issue	FAIR principle	Implications	Recommendations/solutions	Source of FAIR examples
Lack of unique record identifiers (occurrenceID in DwC). The proxy identifier SampleID is not unique in 20% of cases. A composite key is used for mapping to DwC for publication on OBIS, but this is not unique in 30% of cases	Findable, Accessible, Interoperable, Reusable	Individual records cannot be definitively identified; substantially compromises all data handling and analysis	Incorporate DwC term occurrenceID into environmental data template as a required field; provide clear guidance on usage; undertake QA/QC on data submissions to ensure they include valid unique record identifiers	OBIS and GBIF: all records have occurrenceID (a required field) that is persistent and unique
Large-scale duplication of datasets; estimated to be a quarter of total records surveyed	Interoperable, Reusable	Duplication of data impacts on analysis of biodiversity, e.g. potential reduction in estimates of species richness	Incorporate DwC term datasetName into environmental data template; revise internal versioning procedures for the annual contractor data submissions; provision of a DOI to support traceability	OBIS and GBIF: both include dataset name; all records have a unique identifier (occurrenceID); WoRMS: usage of unique AphiaIDs for scientific names
Taxonomic data–quality issues: usage of unaccepted names; misformatting of taxonomy columns and misspellings (85% of records overall)	Accessible, Interoperable, Reusable	Significant data processing necessary before data can be used in analyses; inaccuracies in taxonomic information could impact analyses utilizing datasets	Address taxonomic data–handling procedures including both taxa recorded as scientific names and those recorded as open nomenclature (i.e. with qualifiers or as temporary names); usage of WoRMS backbone and tools such as taxonMatch to match to accepted names only; usage of WoRMS unique AphiaIDs to resolve taxa names means duplicate names (synonyms, homonyms etc) are distinguished	OBIS (in GBIF, issues with taxonomic data handling were identified, i.e. usage of unaccepted names) WoRMS provides gold standard for taxonomic information and is the backbone for OBIS
Data standards not used in DeepData. While DwC used in preparing data for harvesting by OBIS, mismapping of data to DwC terms has occurred	Accessible, Interoperable, Reusable	Database export file not standardized; necessitates significant data processing	Make environmental data template fully DwC compliant; publish data as DwC; revise DwC mapping as currently done for the OBIS ISA node, so that data are treated consistently	OBIS; GBIF; PANGAEA
Bathymetric data unavailable	Reusable	Key information for environmental studies is currently inaccessible	Publish data: begin with pipeline for bathymetric data acquisition from contractors; include option to download ‘raster’ data on the DeepData web portal	GEBCO; NOAA-NCEI
Data structure of data export	Accessible	Different data structure in ‘export query’ versus ‘export pivot query’; the former requires significant data processing	Standardize all data exports to output as DwC-A; begin with improved guidance on the website for data export options so that ‘export pivot query’ is clearly default option for biological data	OBIS ISA node: here, data exports are in DwC format

FAIR principles: data are to be findable, accessible, interoperable and reusable (see Wilkinson et al. (2016) (2) and File S7).

### Making environmental data template fully DwC compliant and remapping of all data to DwC

Our key recommendation is that the ISA update the current environmental data template with a DwC compliant version, with all fields (column headings) in DwC format. DwC is a global, community-led, well-established data standard, and DwC terms are clearly understood, with a readily available, easy-to-read reference guide (https://dwc.tdwg.org/terms/). To accompany this, we recommend that rules are also incorporated into the template to ensure required fields (e.g. occurrenceID) are populated. Contractors or other data providers/stakeholders should also be able to submit data as a Darwin Core Archive (DwC-A). The ISA could consider that at a later stage the environmental data template is entirely phased out for a requirement of data submission as a DwC-A, i.e. as is the case for OBIS and GBIF. We acknowledge that the environmental data template is much broader than the biological data covered here, but data standards including within DwC are available to cover the relevant fields, for example the OBIS-ENV-DATA environmental DwC extension (42; also see table in Rabone et al., 20). Usage of data standards could also be applied to geological data. Full utilization of the global standard DwC would benefit both the contractors and the ISA data team, as well as other stakeholders the user community, and would address all the key issues we have identified with the database. Making the template fully DwC compliant would allow the following:

- All the biological fields included in the environmental data template could be mapped to DwC terms with less ambiguity and more precision. Essential terms that are currently absent from the current database export such as occurrenceID and basisOfRecord would be included as a matter of course. As a result, data will adhere to a common global data standard, allowing data to meet criteria of being FAIR.
- Similarly, essential terms for taxonomic identification that are currently absent from the current database export, e.g. scientificName and identificationQualifier, could be included. DwC also includes the terms ‘verbatimScientificName’ and ‘acceptedScientificName’; therefore, the verbatim name as recorded by the contractor and the accepted name as according to WoRMS identified during data validation (if different) could be included, which would allow for data capture of taxonomic versioning.
- The possibility for misinterpretation of the data is reduced if data mapping to DwC is performed by contractors rather than the ISA. With training, contractors will be well equipped to map to DwC terms including those currently misinterpreted by the Secretariat in mapping, e.g. taxonConceptID and basisOfRecord (see Recommendations).
- This would significantly reduce the risk of duplication, as the unique identifier occurrenceID is allocated by the contractor, avoiding issues downstream. ISA allocating identifiers as is currently happening is a major breakpoint in the system. Inclusion of the DwC term ‘datasetName’ in the template and robust versioning of annual submissions would further reduce duplication.
- The database export could be downloaded as a DwC-A (or as a csv file with an xml metadata file). This would standardize the database export file structure and allow for proper metadata recording, improving all aspects of FAIR. Currently, there are two options for export of biological data: ‘export query’ or ‘export pivot query’. These two options, provided to account for different data types, have a different structure (the former requiring restructuring prior to analysis). Full utilization of DwC would allow for interoperability of the data as the data export could be provided as DwC-A (as is standard practice for OBIS and GBIF).
- The data export from DeepData and the OBIS ISA node would be identical. At present, these datasets should contain identical information but differ owing to the different data processing steps, and more critically, because a unique record identifier is absent from the DeepData export, the records cannot be definitively matched. Once the DwC mapping is revised, datasets could be republished on both databases as matching record sets.
- The DeepData export file as DwC would be FAIR and ready for analysis. The current data export from the database requires significant data processing, for example cleaning of taxonomic information. Similarly significant processing was required for data downloaded via the OBIS ISA node (see Results). With correct implementation and interpretation of DwC terms according to established guidelines, in combination with adjustments to ISA workflows as detailed in the following section, the output from both DeepData and the OBIS ISA node would be ready for analysis.
- Making the template fully DwC compliant would allow the ISA to implement the data processing steps currently done by the OBIS secretariat. It could also facilitate the potential automation of the whole submission process and initial QA/QC steps at a later phase of the database, which could benefit both the ISA and data providers.

### Data management considerations

#### DwC and usage of identifiers

**Figure 4.**
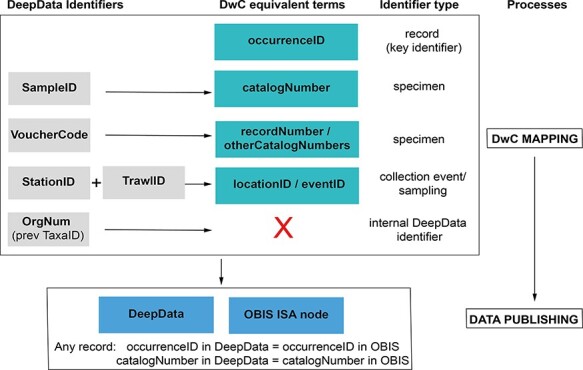
Identifier fields in DeepData, and recommended revision of usage and mapping to equivalent DwC terms. Currently, there is no unique record identifier (occurrenceID) in DeepData or a requirement to include one in the current (2022) environmental data template. This key identifier is needed for the data template, database export and within the database itself. SampleID, a specimen identifier, is currently the key identifier in DeepData and used as a proxy record identifier (although it is neither unique nor persistent) and currently mapped to occurrenceID (as recorded in the ISA DwC guidance; File S5B), but catalogNumber is in fact the equivalent DwC term. VoucherCode instead is currently mapped to catalogNumber but would be correctly mapped to recordNumber (or otherCatalogNumber). Many other non-identifier DeepData fields not shown could be better mapped to DwC terms with less ambiguity and more precision, for example ‘Morphotype’ in the template replaced with ‘taxonConceptID’ (see Recommendations: Darwin Core and usage of identifiers).

We also recommend some key adjustments to data management in the following section to complement the aforementioned process, to address the issues identified and to facilitate republishing of these data. First, fully utilizing DwC would also necessitate a revision in the usage of identifiers (Figure 4). Having datasets with valid unique identifiers is essential and would greatly reduce or even remove duplication. Currently, there is no requirement for a record identifier (occurrenceID in DwC) or one present in DeepData (persistent/unique or otherwise), and it is crucial to address this. In DeepData, the specimen identifier SampleID is used as the record identifier (including within a composite key to generate a unique identifier for DwC mapping for harvesting of data by OBIS). This is problematic for several reasons. First, this identifier is often missing from contractor data submissions, or it is not unique. Second, given that not all environmental data submissions will be individual specimen-level records, it is not appropriate to utilize it as a proxy ‘universal’ record identifier. Third, good data practice requires that any digital record should have its own unique identifier as a matter of course, as this is crucial to any data handling. In fact, occurrenceID is the sole required field in a DwC data submission to OBIS or GBIF. It should be unique and meet criteria of persistence, resolvability, discoverability and authority, for example a globally unique identifier (GUID) (25, 43; see List of Terms). For examples of usage in the CCZ, see Wiklund et al. (44). The ISA allocating identifiers is a key fragility in the system; allocation of unique IDs by contractors would avoid these problems and also mean that the Secretariat would not have to generate a composite key.

Separate specimen identifiers (catalogNumber) are not required in OBIS or GBIF but can support traceability of physical specimens within an institute. The same identifier (i.e. occurrenceID) may be used by some institutes as catalogNumber. However, many collection institutes have a different code (sometimes human readable), and these are used as an internal institutional identifier including physical specimen labels (Rabone et al., Accepted; 45). In the ISA DwC mapping guidance, SampleID has been mapped to occurrenceID, and VoucherCode has been mapped to catalogNumber (File S5A, B), but this is a misinterpretation of the terms, rather the specimen identifier SampleID maps to catalogNumber, and the secondary specimen identifier VoucherCode could be mapped to either of the DwC terms otherCatalogNumber or recordNumber (Figure 4).

Usage of sampling event and location identifiers could also be revised. The DeepData sampling event identifiers StationID and TrawlID are included in the template, but these do not allow for recording of different deployments/samplings within a station for example. This is a non-trivial issue as accurate delineation of samples is key in biodiversity analyses. Here, the DwC terms locationID and eventID could be utilized (Figure 4). An additional identifier that could be included in the database export is the DwC term associatedSequences to capture INSDC accession numbers, unique identifiers within the INSDC system. For data publishing, revising existing usage of identifiers in DeepData, in particular incorporating occurrenceID, would allow records in DeepData and the OBIS to be reconciled: any given record in DeepData would have a persistent record identifier ‘occurrenceID’, and the corresponding record in the OBIS would have the same occurrenceID (Figure 4). Similarly, catalogNumber for the same record in DeepData if present would match the corresponding OBIS record (as would all data fields). Given the centrality of identifiers in data handling, datasets missing unique record identifiers and specimen identifiers where applicable (i.e. occurrenceID and catalogNumber) should be sent back to the data provider/contractor for revision. Guidelines on best practices in usage of identifiers () could be provided by the Secretariat and included in the workshop (section 5.2)

#### Revision of data mapping to DwC

To accompany this process, we recommend comprehensive field (re)mapping to DwC for the environmental data template itself and existing data holdings. For the latter, this revision of data mapping to DwC could be done both for both data submitted via the template, and legacy—or pre-template data. The existing DwC data mapping is incomplete, and incorrect in a few cases (e.g. morphospecies names mapped to taxonRemarks rather than taxonConceptID). It is important to note that because of the current mishandling of taxonomic data, unsupported scientific conclusions could be drawn without full cleaning and interrogation of the data. More comprehensive mapping will also result in better data capture. For example, some contractors have included non-specimen records, such as imagery-based records in the datasets, which could be described using the basisOfRecord field. While a key to mapping template column headings to DwC is provided, this is somewhat buried in the guidance (File S4B). This documentation could be updated once the mapping is revised. Updates for both the general user guide (published version 2018) and the DwC mapping documentation would be beneficial. The user manual for DeepData that is currently available on the website could also be reconfigured to be made more general as the current version is almost entirely focused on data providers.

Data mapping to DwC would also allow for publishing of legacy datasets. This is particularly important given the lack of legacy data available, with very few CCZ studies for example published prior to 2000, as ascertained in the parallel study (39; Rabone et al., Accepted). Although data quality can be highly variable in legacy data, here DeepData could draw on lessons from natural history collections, publishing data with data quality/data completeness flags as done in GBIF for example. The remapping should be done as a batch process with reference to DwC guidance so that datasets are treated consistently. Consultation with the OBIS Secretariat would support this process, given their extensive experience and insight into DwC and biological/environmental data in general. Adjustments to the data processing pipeline may also be required to avoid taxonomic mismatches such as in the *Monticellina* example detailed in Results. This could be achieved by additional scripting in the case of ambiguous taxonomic matches, e.g. where a name matches more than one in the WoRMS database, the higher taxonomy levels are interrogated and cross-referenced.

#### Address record duplication in DeepData

It is important for the Secretariat to prioritize the removal of duplicate records in the database as this can impact diversity estimates in any usage of the datasets. Analysis from the parallel study has shown that the duplicates result in significantly reduced diversity estimates (39; Rabone et al., Accepted). As mentioned above, there is the possibility of erroneous scientific conclusions if the datasets are used in secondary analysis in their current state. Therefore, it is important to make changes to data management, both in usage of identifiers and also at the dataset level. The DwC term datasetName should be included in the template as a required field with guidance provided. Improved versioning and documentation of datasets will assist both in preventing and identifying duplication. Communication and involvement of the contractors will facilitate this process. Contractors could also be required to do iterative data reporting rather than one-off submissions where applicable, i.e. every year the entire dataset, along with any additional new records are submitted, and no ‘one-off’ data submissions are made. This would ensure that year on year, changes to records are captured, e.g. updates to taxonomic identifications, and potential for harvesting of duplicate datasets is minimized. We recommend that changes are also made to the ISA data-publishing strategy, so that rather than publishing contractor data received from 2015 up to the present, the reverse is applied, i.e. the latest data submissions post-QA/QC are published. Any additional data from previous year’s submissions not included in a current submission, e.g. contracts that are no longer active are then added. This will again reduce potential duplication. Further, once record identifiers are incorporated into the template itself, i.e. occurrenceID (Figure 4), any duplicates at the record level could be automatically flagged for example through cross-referencing of these identifiers during the data submission process.

### Consultation and training workshops with contractors and the scientific community

To support the DwC submission process, training and workshops for contractors, also involving the scientific community and other stakeholders, could be considered by the ISA. Wider involvement of the scientific community is important, both for user feedback on the database and to broaden the data-provider base and encourage publication of non-contractor data on DeepData. The workshops could focus on the relevant databases, tools and data standards: in particular, OBIS, WoRMS and DwC. There are online tools available, which could be utilized in the workshop. These include the WoRMS taxon match tool to help with taxonomic data validation (Table 2), the GBIF DwC assistant and validator and the Integrated Publishing Toolkit (46) to support mapping datasets to DwC.

As missing information in the database is often a result of incomplete contractor data submissions, this could be addressed in a combination of training, consultation with the contractors, documentation and incorporating rules in the template so that mandatory fields (e.g. occurrenceID) have to be (correctly) populated to submit the data. Key information for biological/ecological studies was often absent from datasets, for example relative density and abundance data, depth, sampling method, taxa identification method, habitat (e.g. nodule/sediment/water column) and broad habitat classification (e.g. ‘seamount’/‘abyssal plain’/‘rocky outcrop’). These are important data both for deep-sea research and for developing environmental policy both for the region and at broader spatial scales. Establishing a line of communication with the contractors could help address some of these data gaps and wider data-quality issues. Together with the DwC submission process and additional QA/QC, this could result in greater quality of submitted data to be ingested into the database, with fewer processing steps required, to the benefit of all stakeholders. A general emphasis should be on the quality rather than quantity of data. While issues remain outstanding, the ISA could consider documenting database limitations and data gaps clearly on their website to inform end users (including policymakers) before they conduct any analyses.

### Potential future developments of DeepData

As DeepData reaches a more mature state, further developments of DeepData would be worthwhile. Our review has focused in the main part on the data quality of the biological database output; here, we turn to web functionality. It should be noted however, that as web functionality is inherent to general usability and user experience, it is a key element of general database functionality. Also some of the recommendations listed later, in particular provision of bathymetric data, will be critical to characterizing deep-sea environments and therefore should not necessarily be regarded as ‘optional extras’ but rather as core development. Extensive testing of the web interface is recommended. With data systems, usability and user testing is more critical than theories as to how the systems may work. The ISA here could draw on the model of ‘agile’ software development with extensive user testing and response to user feedback (Rabone & Glover, in review; 47). These developments may also require additional funding. There is a clear case to be made for increased resourcing of DeepData, given the importance, complexity and scale of the database and its potential as a decision-making tool for environmental management. This reflects a wider issue in resourcing of science databases where the fragility of database funding mechanisms belies their key importance in research (20). While additional developments are covered here, this is not intended to be comprehensive and the ISA could encourage the user community to provide wider feedback.

- Provision of an application programming interface (API) to allow the database to be directly interrogated. This will be a most useful tool for utilizing the database and improve the accessibility of data.
- Provision of a digital object identifier (DOI) from DeepData to allow citation of datasets, as currently available for OBIS and GBIF. This would also allow for versioning and traceability as well as data citation.
- Move to a web-based data submission platform, where the DwC-A is submitted via the website, and automated QA/QC checks are initiated, e.g. files submitted without valid identifiers could generate an error code as is currently done on web forms.
- Provide information on database and data updates, e.g. when the database has been updated and a list of datasets published. This will support FAIRness of data and general transparency (34, 48, 49). This is currently listed on the website as an upcoming feature (i.e. publication of a file catalogue: “for clarity and transparency purposes, the ISA Secretariat will publish a file catalogue on regular basis, listing all publicly available data files contained in DeepData”; https://www.isa.org.jm/deepdata/about#block-seabed-page-title). This would be straightforward to implement. It could also include a list of submitted datasets that are yet to be published, therefore clarifying which contractors are actively collecting data (Table 1).
- Provide a dynamically updated cruise inventory on the database for all cruises that have taken place up to current cruises and potentially those in planning. This could be very basic with research vessel and contractor name/s, with cruise dates (e.g. Table 1), but would be very helpful information for all stakeholders. This could even provide a model for the cruise notification system proposed in the Biodiversity Beyond National Jurisdiction (BBNJ) treaty text (20).
- The functionality to interrogate data by the APEI layer—currently any data outside a contract or reserved area is labelled as ‘OA’ (outside area) rather than with the APEI in question. This requires geographic mapping of records (e.g. in R or QGIS) to ascertain the actual record location. The usability of the web interface could be developed further, for example the ability to click on a section of the map, such as a given contract area or APEI, and a summary of available data for that given region is made visible in a side-bar. Such functionality is not duplicating what is present on the OBIS ISA node and is aligned with the GIS–based focus of DeepData.
- Web functionality whereby taxonomic experts can flag erroneous identifications in records on the web portal, as ‘community curation’. This is possible in both WoRMS and GBIF (via different mechanisms), e.g. in WoRMS, taxonomic editors can add or edit records. As similar functionality is planned in OBIS, when this feature is live in OBIS, potentially the ISA data team could be alerted to any tagged records via the OBIS ISA node. This could allow for simple errors to be identified, such as pelagic species recorded as benthic. As a wider point, a pipeline to identify pelagic taxa recorded as collected from benthic samples, found to be extensive in DeepData (Rabone & Glover, in review; 39; Rabone et al., Accepted), would be of great benefit and could be considered as an additional taxonomy QA/QC step, e.g. the cleaned taxa names compared to attribute data in WoRMS and pelagic species named could be tagged.
- Development of a data dashboard on DeepData for interrogating, summarizing and visualizing the data. The emphasis should be on making the dashboard as simple as possible. Now that data are available on the OBIS ISA node, where there is significant functionality for summarizing and visualizing data, there may not be the same imperative to develop this element of the web interface. However, different databases have different user communities; and some stakeholders are likely to use DeepData only and not the OBIS ISA node. This dashboard may be particularly helpful for policymakers, who may be less likely to download and analyse the database holdings. It will also support FAIRness of the data and general transparency.

An additional improvement for DeepData could include the storage of relevant literature, straightforward given existing functionality to store documentation (‘Docs’ tab on the website; Figure S1). Our parallel study shows key data gaps in DeepData where information is available in the literature (39; Rabone & Glover, in review; Rabone et al., Accepted). This would also align with the ISA mandate to facilitate and support marine scientific research in the Area. Similarly, DeepData could include storage and handling of image data, for example megafauna specimen imagery or *in situ* seabed images. Again with the ‘Photo’/‘Video Gallery’ tab in DeepData, the functionality to store and publish imagery is already in place. There is a precedent here too with the CCFZ image atlas for *in situ* imagery, ‘Atlas of Abyssal Megafauna Morphotypes of the Clipperton-Clarion Fracture Zone’ co-administered by the ISA, which was in wide usage by researchers (e.g. 50). However, image data are computationally expensive in terms of required storage and the technicalities of handling more complex data types. DeepData could potentially partner with platforms such as Bio-Image Indexing and Graphical Labelling Environment (BIIGLE) (51) to provide images with metadata to develop image libraries. The mechanics of how this partnership could work in practice may need some thought as image annotation platforms like BIIGLE do not tend to specialize in storing imagery, but there is a clear need for such functionality. Databases of imagery with quality metadata could support machine learning identification efforts, as currently done with iNaturalist, a global citizen science application for recording species observations (https://www.inaturalist.org/).

This could be extended to acoustic images, e.g. multibeam imagery for bathymetry. Bathymetric data are listed on the database but not available other than a few bathymetric metadata records for a sole contractor. Given the categorization of data into ‘Point’ and ‘Line’ on the database, a category for ‘raster’ or similar should be added to allow for bathymetric data, which are typically in this format. Bathymetry is the first dataset collected in deep-sea surveys and essential to ecological studies. As it is important for these data to be made available, here the ISA could make the provision of bathymetry from all offshore campaigns a requirement and develop a pipeline for publication of these data. DeepData could also work directly with the Multibeam Bathymetry Database (MBBDB) supported by the NOAA (National Oceanic and Atmospheric Administration) [NOAA National Centers for Environmental Information. 2004: Multibeam Bathymetry Database (MBBDB). Available at https://doi.org/doi:10.7289/V56T0JNC]. Also the ISA could work with The General Bathymetric Chart of the Oceans (GEBCO)–Nippon Foundation SeaBed 2030 Project (52), where, as for WoRMS and OBIS, existing partnerships are in place.

## Conclusions and future directions

While our study is focused on the CCZ and the ISA database, it illustrates some of the wider challenges, and opportunities for biodiversity databases, in particular for developing their utility for both research and environmental policy. The DeepData collaborations with OBIS and WoRMS and data mapping to DwC herald a very welcome new phase and a rapid evolution for the database and ISA data management practices. DeepData is now integrating with global databases and global common data standards, allowing for data exchange and integration, for data to be FAIR. However, non-trivial issues with data quality remain, particularly regarding identifiers, duplication and treatment of taxonomic information. Our review of the database has illustrated the integral importance of global community-led data standards and persistent identifiers for data. These challenges of DeepData reflect those in the wider biodiversity data ecosystem. However, given the direct connection of the database with the regulator, and the possibility it may be directly utilized in development of environmental policy for a potential extractive industry, it is even more urgent that these issues are addressed. There is potential for DeepData to provide an invaluable resource both for research and environmental management. It would be of great value for example to be able to directly interrogate the database for species diversity or distribution. Or on a regional scale, DeepData could ultimately become critical in helping to develop the REMP for the CCZ and other seabed regions managed by the ISA. While feedback from user communities of databases via feature requests or bug-tracking for example is a common practice, more formal and comprehensive assessments of databases like the current study are rare, and we hope in the process to have provided the ISA with useful and implementable recommendations. The database is at a nascent phase of its development; here, engagement and involvement of the science community, policymakers and contractors to further the development of DeepData is critical. There is a collective responsibility amongst all stakeholders to support open data efforts such as DeepData and community data curation. The ISA is well placed to lead and co-ordinate activities and encourage efforts in best practice and eventually may even in time provide an exemplar for high-quality deep-sea biological datasets. Such information could be utilized for biodiversity assessments and observing programmes, including contributions to indicators and variables such as EOVs and EBVs (3, 4). These could be applied at regional scales, with DeepData contributing information to the proposed Deep Ocean Observing Strategy (DOOS) demonstration project for the CCZ (53) and even at global scales across ocean basins. In time, DeepData may be viewed not through a CCZ or even an ISA lens but rather through a global one and as an integral part of the global data landscape. Partnerships with international science frameworks and programs including the UN Decade of Ocean Science, DOOS, major genomic data projects like Earth Biogenomes (54) and the GEBCO Seabed 2030 mapping programme (among others) will be crucial, as will integration into the wider policy landscape, i.e. the UN BBNJ treaty process. An ultimate focus on the importance of biodiversity data to support conservation efforts is key.

## Supplementary Material

baad013_Supp

## Data Availability

All datasets and code are available as supplementary files and archived on GitHub (https://github.com/howlerMoonkey/). This paper is part of a larger study, and the parallel paper is available as a preprint (First Synthesis of Metazoan Biodiversity in the World’s Largest Mineral Exploration Frontier. http://dx.doi.org/10.2139/ssrn.4276976). Both studies originate from the following report: Rabone, M., Glover, A.G., 2022. A review and synthesis of CCZ benthic metazoan biodiversity data from the ISA DeepData database, the literature and other published sources. Prepared for The Pew Charitable Trusts. Report number NHM SON20001/PewFR.

## List of terms

(terms in bold are DwC terms)

**basisOfRecord**: a DwC term to describe record type (‘the specific nature of the data record’), e.g. preservedSpecimen

Checklist: an inventory of species/taxa names, often organized by taxonomic group or region

**catalogNumber**: a DwC term for specimen identifier [TDWG definition: ‘an identifier (preferably unique) for the record within the data set or collection’]

Contractors: holders of mineral exploration contracts

DwC: Darwin Core, a global data standard administered by the TDWG

Identification qualifier: taxonomic identification qualifiers, such as aff., cf., sp. and nov., to designate a species in ‘open nomenclature’, [**identificationQualifier** in DwC terminology; TDWG definition: ‘a brief phrase or a standard term (“cf.”, “aff.”) to express the determiner’s doubts about the Identification’]

MOTUs: Molecular Operational Taxonomic Units; a type of informal or temporary species name (open nomenclature)

Morphospecies: informal species names used prior to formal description (open nomenclature). Other terms for informal species names include morphotypes, M/OTUs, working species or temporary names.

**occurrenceID**: a DwC term for record identifier [TDWG definition: ‘an identifier for the Occurrence (as opposed to a particular digital record of the occurrence). In the absence of a persistent global unique identifier, construct one from a combination of identifiers in the record that will most closely make the occurrenceID globally unique’]

Occurrence data: distributional records of species/taxa

Open nomenclature: system of signs to describe uncertainty around identifications or designate informal/temporary taxa names prior to formal description (e.g. morphospecies)

Scientific name: the designation or identification of an organism, **scientificName** in DwC terminology (TDWG definition: ‘the full scientific name, with authorship and date information if known. When forming part of an Identification, this should be the name in the lowest-level taxonomic rank that can be determined. This term should not contain identification qualifications, which should instead be supplied in the IdentificationQualifier term’)

**taxonConceptID**: a DwC term for open nomenclature—temporary/informal species names (‘an identifier for the taxonomic concept to which the record refers, not for the nomenclatural details of a taxon’)
