# Supplementary material for: A review of the International Seabed Authority database DeepData from a biological perspective: challenges and opportunities in the UN Ocean Decade
Source: Database (Oxford). 2023 Mar 30;2023:baad013. doi: 10.1093/database/baad013 (PMC10064262; doi:10.1093/database/baad013)

International Seabed Authority

20-Nov-2020

Deep Data Correlation to Darwin Core in the Context of OBIS

This document outlines the correlation between Deep Data and Darwin Core for OBIS Integration. What is Darwin Core? According to https://dwc.tdwg.org/terms/ **Darwin Core is a body of standards for biodiversity informatics. It provides stable terms and vocabularies for sharing biodiversity data. Darwin Core is maintained by TDWG (Biodiversity Information Standards, formerly The International Working Group on Taxonomic Databases).**

Deep Data

From deep data a number of structures were selected based on the definition of DwC Terms. These represent formatted input data submitted by Contractors for data on Biological samples collected at specific points or Biological Samples collected on a trawl

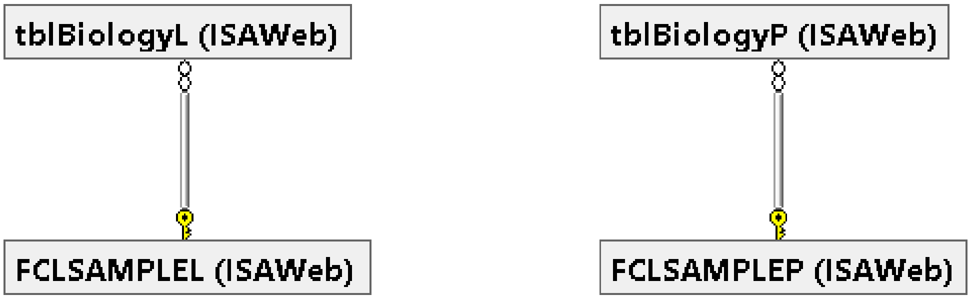

DeepData Mapping Fields

Key mapping fields are taken from the ANALYSIS data.

Category	Analysis
Ecology	Nominal size Category
Ecology	Number of individuals
Ecology	Relative abundance (%)
Ecology	Relative dominance (%)
Ecology	Species density - benthos (ind/m2)

Ecology	Species density - pelagic (ind/m3)
Ecology	Total Biomass collected (g/m2)
Organism Details	Sex
Taxonomist information	Taxonomist
Taxonomist information	Taxonomist E-mail
Taxonomist information	Taxonomist Institution
Taxonomy ID	Species
Taxonomy ID	Order
Taxonomy ID	Phylum
Taxonomy ID	Class
Taxonomy ID	Family
Taxonomy ID	Genus
Taxonomy ID	Identification Method
Taxonomy ID	Kingdom
Taxonomy ID	Morphotype
Taxonomy information	Database Taxa ID
Taxonomy information	Description of DNA Sequence
Taxonomy information	Notes on taxanomic identification
Taxonomy information	Putative species name or number
Taxonomy information	Voucher code
Taxonomy information	Taxonomic Database
Taxonomy information	Taxonomic Status

Darwin Core terms

DwC terms correspond to the column names of the dataset. A list of all possible Darwin Core terms can be found on TDWG. Below is an overview of the most relevant Darwin Core terms to consider when contributing to OBIS, with guidelines regarding their use.

Note that OBIS currently has eight required DwC terms: `occurrenceID`, `eventDate`, `decimalLongitude`, `decimalLatitude`, `scientificName`, `scientificNameID`, `occurrenceStatus`, `basisOfRecord`.

Correlation Guidelines

ISA DeepData will not map all DwC Terms but will aim to provide as much comprehensive mapping as is available. Data currently unavailable with no relative mapping will be removed from final record dump provided to OBIS

Occurrence

DwC Term	Deep Data Field
occurrenceID	Concatenation of TrawlID/StationID & SampleID
catelogNumber	Concatenation of Catalogue_OID & SampleID
RecordedBy	Concatenation of Taxonomist +','+Taxonomist E-mail
individualCount	Number of Individuals

organismQuantity	On condition if Species density has a value else Number of Individuals
organismQuantityType	In case of Species density then value: ‘indivdual density per metre cube’ else ‘individuals’
sex	sex
occurenceStatus	‘present’
associatedSequences	Description of DNA Sequence
occurrenceRemarks	Remarks

Event

DwC Term	Deep Data Field
eventID	Concatenation of Cruise leg and sampleID
eventDate	SampleDateStart/SampleDate
eventTime	Time part of SampleDate
month	Month part of SampleDate
day	Day part of SampleDate
Habitat	HabitatDescription
samplingProtocol	SamplingDevice

Location

DwC Term	Deep Data Field
locationId	Concatenation of Station/TrawlID and AreaKey
MinimumDepthInMeters	minDepth
MaximumDepthInMeters	maxDepth
vermatimDepth	MinDepth and MaxDepth
decimalLatitude	StartLatidude
decimalLongitude	StartLongitude
verbatimCoordinateSystem	‘decimal degrees’
verbatimSRS	‘WGS84’

Identification

DwC Term	Deep Data Field
identificationID	Concatenation of CruiseLeg and TaxonID
typeStatus	Taxonomic Status
identifiedBy	Taxonomist
SampleDateStart	dateIdentified
identificationVerificationStatus	0
typeStatus	Voucher status

Taxon

DwC Term	Deep Data Field
taxonID	Concatenation of Cruise leg and SampleID
scientificName	Species or Genus
scientificNameID	Putative species name or number
Kingdom	Kingdom
Phylum	Phylum
class	Class
order	Order
family	Family
genus	Genus
taxonomicStatus	Taxonomic Status

RecordLevel

DwC Term	Deep Data Field
type	‘Event’
license	Attribution 3.0 IGO (CC BY 3.0 IGO)
rightsHolder	Contractor
accessRights	Terms of use DeepData
bibliographicCitation	‘International Seabed Authority, DeepData’
institutionID	Interiational Seabed Authority
basisOfRecord	‘Taxon’

In addition to the above mapping, data such as relative abundance is added to the MeasurementOrFact DwC standard. This can be extended to any variable in DeepData that can be represented.

MeasurementOrFact

DwC Term
measurementID
measurementType
measurementValue
measurementUnit

Data Cleaning

A number of steps were taken to cleanse the data provided by contractors in order to improve Taxon Matching on OBIS. This involved removing temporary names e.g. sp. and remap to

File Transfer Protocol.

In providing data to OBIS, DeepData will be sending a single file per month of the complete dataset. Which will be picked up by OBIS Technical team and formatted for presentation on OBIS Platform.

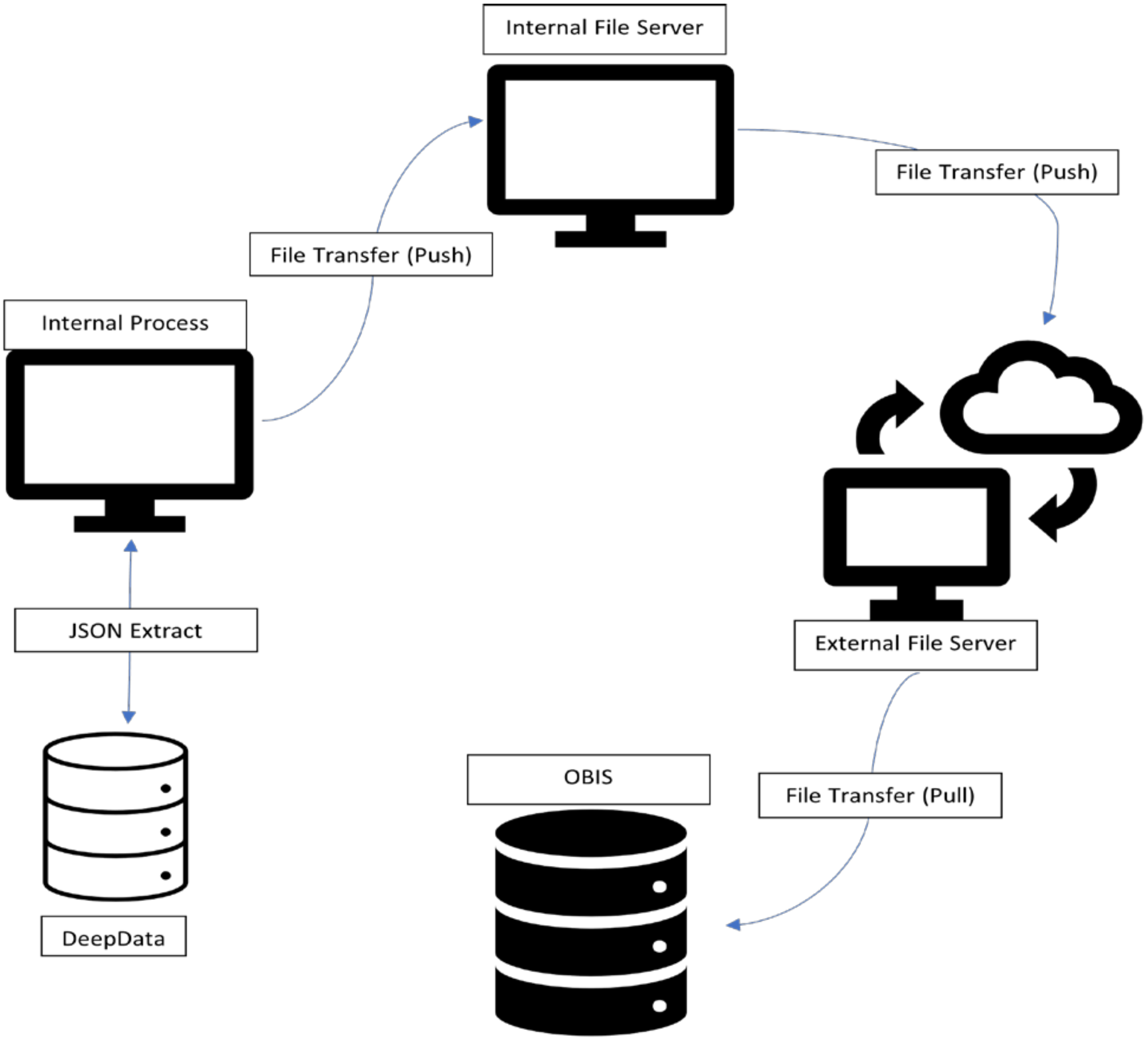

Supplement: baad013_Supp [file baad013_supp.zip › SF5A_Deep Data Darwin Core Mapping 2.pdf]
