# Supplementary material for: A review of the International Seabed Authority database DeepData from a biological perspective: challenges and opportunities in the UN Ocean Decade
Source: Database (Oxford). 2023 Mar 30;2023:baad013. doi: 10.1093/database/baad013 (PMC10064262; doi:10.1093/database/baad013)
Supplement: baad013_Supp [file baad013_supp.zip › SF7_FAIR_prinicples_box2_in_Wilkinson_etal_2016.docx]

The FAIR Guiding Principles (reproduced from Box 2 in Wilkinson et al., (2016)

To be Findable:

F1. (meta)data are assigned a globally unique and persistent identifier

F2. data are described with rich metadata (defined by R1 below)

F3. metadata clearly and explicitly include the identifier of the data it describes

F4. (meta)data are registered or indexed in a searchable resource

To be Accessible:

A1. (meta)data are retrievable by their identifier using a standardized communications protocol

A1.1 the protocol is open, free, and universally implementable

A1.2 the protocol allows for an authentication and authorization procedure, where necessary

A2. metadata are accessible, even when the data are no longer available

To be Interoperable:

I1. (meta)data use a formal, accessible, shared, and broadly applicable language for knowledge representation.

I2. (meta)data use vocabularies that follow FAIR principles

I3. (meta)data include qualified references to other (meta)data

To be Reusable:

R1. meta(data) are richly described with a plurality of accurate and relevant attributes

R1.1. (meta)data are released with a clear and accessible data usage license

R1.2. (meta)data are associated with detailed provenance

R1.3. (meta)data meet domain-relevant community standards
